# Development and validation of a nomogram model of depression and sleep disorders and the risk of disease progression in patients with breast cancer

**DOI:** 10.1186/s12905-024-03222-9

**Published:** 2024-07-03

**Authors:** Jun Shen, Dan Zhou, Meng Wang, Fan Li, Huan-Huan Yan, Jun Zhou, Wen-Wen Sun

**Affiliations:** Department of Breast surgery, The First People’s Hospital of LianYunGang, No.6 Zhenhua East Road, High-tech Square, LianYunGang, 222002 Jiangsu Province China

**Keywords:** Depression, Progressive disease, Risk prediction model, Sleep disorder

## Abstract

**Background:**

In this study, we investigated the relationship between the risk of postoperative progressive disease (PD) in breast cancer and depression and sleep disorders in order to develop and validate a suitable risk prevention model.

**Methods:**

A total of 750 postoperative patients with breast cancer were selected from the First People’s Hospital of LianYunGang, and the indices of two groups (an event group and a non-event group) were compared to develop and validate a risk prediction model. The relationship between depression, sleep disorders, and PD events was investigated using the follow-up data of the 750 patients.

**Results:**

SAS, SDS, and AIS scores differed in the group of patients who experienced postoperative disease progression versus those who did not; the differences were statistically significant and the ability to differentiate prognosis was high. The area under the receiver operating characteristic (ROC) curves (AUC) were: 0.8049 (0.7685–0.8613), 0.768 (0.727–0.809), and 0.7661 (0.724-–0.808), with cut-off values of 43.5, 48.5, and 4.5, respectively. Significant variables were screened by single-factor analysis and multi-factor analysis to create model 1, by lasso regression and cross-lasso regression analysis to create model 2, by random forest calculation method to create model 3, by stepwise regression method (backward method) to create model 4, and by including all variables for Cox regression to include significant variables to create model 5. The AUC of model 2 was 0.883 (0.848–0.918) and 0.937 (0.893–0.981) in the training set and validation set, respectively. The clinical efficacy of the model was evaluated using decision curve analysis and clinical impact curve, and then the model 2 variables were transformed into scores, which were validated in two datasets, the training and validation sets, with AUCs of 0.884 (0.848–0.919) and 0.885 (0.818–0.951), respectively.

**Conclusion:**

We established and verified a model including SAS, SDS and AIS to predict the prognosis of breast cancer patients, and simplified it by scoring, making it convenient for clinical use, providing a theoretical basis for precise intervention in these patients. However, further research is needed to verify the generalization ability of our model.

**Supplementary Information:**

The online version contains supplementary material available at 10.1186/s12905-024-03222-9.

## Background

Breast cancer is the most prevalent malignant tumor and the primary cause of malignant tumor-related mortality among women [1]. It accounts for approximately 25% of malignant tumors and 15% of tumor-related mortality among women [2]. Breast cancer has the highest annual growth rate of any malignant tumor at 2.98%, and its incidence rate is steadily on the rise in the United States [3]. In China, the incidence of breast cancer has surpassed lung cancer, and it ranks first among all malignant tumors among women.

With the intensification of the biopsychosocial medicine model, medical professionals have focused on the impact of unhealthy emotional factors on the diagnosis, treatment, and prognosis of patients with cancer, particularly breast cancer [4, 5]. Studies indicate that patients with breast cancer experience a variety of unhealthy emotional manifestations, including anxiety and depression [6], self-abasement, concerns about daily life, and negative attitudes towards life [7]. The prognosis of patients is directly influenced by psychological abnormalities, and about 60% of patients with metastatic breast cancer have sleep disorders, anxiety, and depression [8]. The mortality rate for patients with breast cancer whose disease is complicated by depression is higher than 19%. Sleep disorders are also significant in patients with cancer, with an incidence of about 30–80% [9]. Sleep disorders frequently impair vital biological processes like immunity.

Breast cancer is a serious threat to female health and has a psychological impact on patients, causing depression and sleep disorders, leading to a poor prognosis [10]. In order to provide accurately targeted interventions, additional research is required to determine how to develop a prediction model for breast cancer progression based on depression and sleep disorders.

## Materials and methods

### Participants

In this retrospectively, case control study, 750 patients with breast cancer who received surgical treatment in Lianyungang First People’s Hospital between January 1, 2015 and December 1, 2018 were retrospectively selected. All patients had clear outcomes at the follow-up cutoff point and were divided into two groups with and without disease progression. Their outpatient and inpatient treatments were confirmed using the hospital’s electronic medical record system, the medical histories of the patients, the patient follow-up table, and return telephone consultations. The follow-up period will conclude in June 2020. At this juncture, we define the event group as encompassing disease progression events that occurred prior to this time point, while the censored data refers to instances where no disease progression events were observed before this specific time point.

The inclusion criteria were as follows: ① surgical patients who were pathologically diagnosed with breast cancer; ② patients who had not received any other treatments prior to surgery; ③ patients older than 18 years of age ④ patients with a Karnofsky Performance Status score > 70; ⑤ patients who could communicate and interact effectively and had sound comprehensive and cognitive abilities; ⑥ patients who provided signed informed consent for this study; and ⑦ patients with complete medical records and follow-up data. The exclusion criteria were as follows: ① patients with an expected survival period of less than six months; ② patients who were complicated with other tumors or who had previously suffered from other tumors; ③ patients who had comorbidities of severe heart, liver, or kidney diseases; ④ lactating and pregnant women; and ⑤ patients who were unable to communicate effectively or did not cooperate with follow-ups. We defined an event of disease progression (endpoint event) with specific criteria: (1) Soft Tissue Metastasis and Recurrence: This includes soft tissue metastasis and recurrence, encompassing lymph nodes and chest wall soft tissues. Diagnosis by biopsy pathology must confirm either recurrence or metastasis (excluding primary cancers on the contralateral side). (2) Liver and Lung Metastasis: Confirmation is through pathology. (3) Bone Metastasis: Confirmation is through pathology, or experts determine bone metastasis through methods such as ECT along with X-Ray/CT/MRI (Whether the patient is revisited regularly or is further examined for symptoms of bone pain, once abnormalities are present on X-Ray/CT or MRI, CT includes osteoclastic or osteogenic changes, MRI shows abnormal signals with peripheral soft tissue swelling, or ECT indicates nuclide concentration that cannot be explained by other causes, such as trauma. In this case of suspected bone metastasis, if the patient cannot be clearly punctured, it is necessary to consult with relevant experts, including nuclear medicine, orthopedics, and imaging departments, to confirm bone metastasis.). (4) Brain Metastasis: Confirmation is through imaging diagnosis. Brain metastasis is considered if accompanied by relevant symptoms such as headaches and high intracranial pressure. (5) Bone Marrow Metastasis: Confirmation is through bone marrow biopsy pathology. (6) Metastases from Other Parts: Confirmation is through pathology or PET-CT scan.

### Observation indices

The baseline characteristics and follow-up data of the patients were extracted from the medical record data system of our hospital. We collected the name, age (in years), income, surgical method, tumor/number/metastasis (TNM) stage, marital status, family support, education, religion, self-rating anxiety scale (SAS) score, self-rating depression scale (SDS) score, Athens insomnia scale (AIS) score, prognosis indicators, and follow-up time of the patients. The income category included high (> RMB 4,000/month) and low income (< RMB 4,000/month); surgical methods included modified radical mastectomy and breast-conserving surgery; TNM (tumor, node, metastasis) staging was determined based on the 2014 National Comprehensive Cancer Network (NCCN) guidelines; marital status included being married, unmarried, divorced, and widowed; family support included “good” and “deficient” (as reported by the patients); religion was classified as “yes” or “no,” and SAS and SDS were applied [11]. The AIS was used to evaluate sleep disorders [12]. The SAS, SDS, and AIS scores of patients were measured on admission. The prognosis index referred to progressive disease (PD) events, including visceral metastasis, local recurrence, and lymph node metastasis.

### Statistical methods

A *t*-test and Pearson’s chi-squared test were used to analyze the correlation between the clinical characteristics of the patients and their prognoses using R 4.02 software. The Kaplan-Meier survival curve and the receiver operating characteristic (ROC) curve were drawn using GraphPad 8.0 software. Univariate and multivariate COX regression analysis, cross-lasso regression, random forest, and stepwise regression were used to screen variables. Different models are combined by screening variables, and the models are compared and analyzed. All processes were performed by R software using the glmnet (4.1–4), randomForestSRC (3.0.2), and MASS (7.3–56) packages, and *P* < 0.05 indicated statistically significant differences. A nomogram was drawn based on the results of the analysis obtained with the regression model. The area under the ROC curve (AUC) with the subject operating characteristic and the time-dependent ROC curve were used to identify and correct the model, and the correction curve was drawn. The differences between the various models were evaluated using the net reclassification index (NRI). The nomogram was used for evaluating clinical benefit with the decision curve analysis (DCA) and clinical impact curve (CIC). The following R packages were used: pROC (1.180), timeROC (0.4), nricens (1.6), survIDINRI (1.1–1), survC1 (1.0–3), rmda (1.6).

## Results

### Baseline data

The investigation included 750 patients with a mean age of 62 years (7.6 years). The longest follow-up period was 66 months (median: 36 months), and 169 PD events occurred at the conclusion of the follow-up period. The study cohort was divided by a 7:3 ratio into 527 cases in the training set and 223 cases in the validation set, with 126 PDs occurring in the training set and 43 PDs observed in the validation set, as detailed in Appendix 1. There were statistically significant differences (*P* < 0.05) in income, TNM, marital status, family, education, SAS, SDS, and AIS in the cohorts with and without PD events, including the full cohort, training set, and validation set, and there were no significant differences between the three cohorts, as detailed in Table 1.

**Table 1:** Baseline indexes and univariate analysis

	All data set				Train data set				Test data set			
Variable	Overall, *N* = 750^1^	No events, *N* = 581^1^	Events, *N* = 169^1^	*p*-value^2^	Overall, *N* = 527^1^	No events, *N* = 401^1^	Events, *N* = 126^1^	*p*-value^2^	Overall, *N* = 223^1^	No events, *N* = 180^1^	Events, *N* = 43^1^	*p*-value^3^
**Age, Mean(SD)**	62.9(7.6)	63.2(6.8)	61.6(9.9)	0.9	62.6(7.9)	63.2(7.1)	60.8(10.0)	0.3	63.4(6.7)	63.3(6.0)	63.7(9.2)	0.14
**Income, n(%)**				**< 0.001**				**0.002**				**0.021**
Low	394(53%)	328(56%)	66(39%)		261(50%)	214(53%)	47(37%)		133(60%)	114(63%)	19(44%)	
High	356(47%)	253(44%)	103(61%)		266(50%)	187(47%)	79(63%)		90(40%)	66(37%)	24(56%)	
**Operation, n(%)**				0.10				0.4				0.11
MRM	420(56%)	316(54%)	104(62%)		299(57%)	223(56%)	76(60%)		121(54%)	93(52%)	28(65%)	
BCS	330(44%)	265(46%)	65(38%)		228(43%)	178(44%)	50(40%)		102(46%)	87(48%)	15(35%)	
**TNM, n(%)**				**0.003**				**0.012**				0.2
I	304(41%)	252(43%)	52(31%)		205(39%)	168(42%)	37(29%)		99(44%)	84(47%)	15(35%)	
II-III	446(59%)	329(57%)	117(69%)		322(61%)	233(58%)	89(71%)		124(56%)	96(53%)	28(65%)	
**Marital, n(%)**				**< 0.001**				**< 0.001**				**< 0.001**
No spouse	130(17%)	73(13%)	57(34%)		88(17%)	49(12%)	39(31%)		42(19%)	24(13%)	18(42%)	
Married	620(83%)	508(87%)	112(66%)		439(83%)	352(88%)	87(69%)		181(81%)	156(87%)	25(58%)	
**Family, n(%)**				**< 0.001**				**0.001**				**0.002**
Normal	128(17%)	80(14%)	48(28%)		95(18%)	60(15%)	35(28%)		33(15%)	20(11%)	13(30%)	
Good	622(83%)	501(86%)	121(72%)		432(82%)	341(85%)	91(72%)		190(85%)	160(89%)	30(70%)	
**Education, n(%)**				**< 0.001**				**0.002**				**0.031**
Below high school	334(45%)	281(48%)	53(31%)		213(40%)	177(44%)	36(29%)		121(54%)	104(58%)	17(40%)	
High School and above	416(55%)	300(52%)	116(69%)		314(60%)	224(56%)	90(71%)		102(46%)	76(42%)	26(60%)	
**Religion, n(%)**	96(13%)	76(13%)	20(12%)	0.7	73(14%)	57(14%)	16(13%)	0.7	23(10%)	19(11%)	4(9.3%)	> 0.9
**SAS, Mean(SD)**	38.8(9.0)	36.6(8.2)	46.4(7.7)	**< 0.001**	38.9(9.1)	36.7(8.3)	46.1(7.7)	**< 0.001**	38.6(8.9)	36.5(7.9)	47.2(7.8)	**< 0.001**
**SDS, Mean(SD)**	43.7(7.9)	42.0(7.1)	49.6(7.5)	**< 0.001**	43.9(7.7)	42.2(7.1)	49.0(7.3)	**< 0.001**	43.3(8.3)	41.4(7.2)	51.3(7.8)	**< 0.001**
**AIS, Mean(SD)**	4.4(1.5)	4.1(1.3)	5.6(1.5)	**< 0.001**	4.5(1.5)	4.1(1.4)	5.5(1.6)	**< 0.001**	4.4(1.4)	4.0(1.2)	5.9(1.3)	**< 0.001**
**Follow-up time, Median**(**IQR**)	10(6,17)	10(5,17)	10(6,17)	> 0.9	10(6,17)	10(5,18)	10(6,16)	0.8	11(7,17)	12(7,14)	10(7,18)	0.6

^1^Mean(SD) or Median(IQR) or Frequency(%)

^2^Wilcoxon rank sum test; Pearson’s Chi-squared test

^3^Wilcoxon rank sum test; Pearson’s Chi-squared test; Fisher’s exact test

### SAS, SDS, AIS, and prognosis

**Fig. 1:**
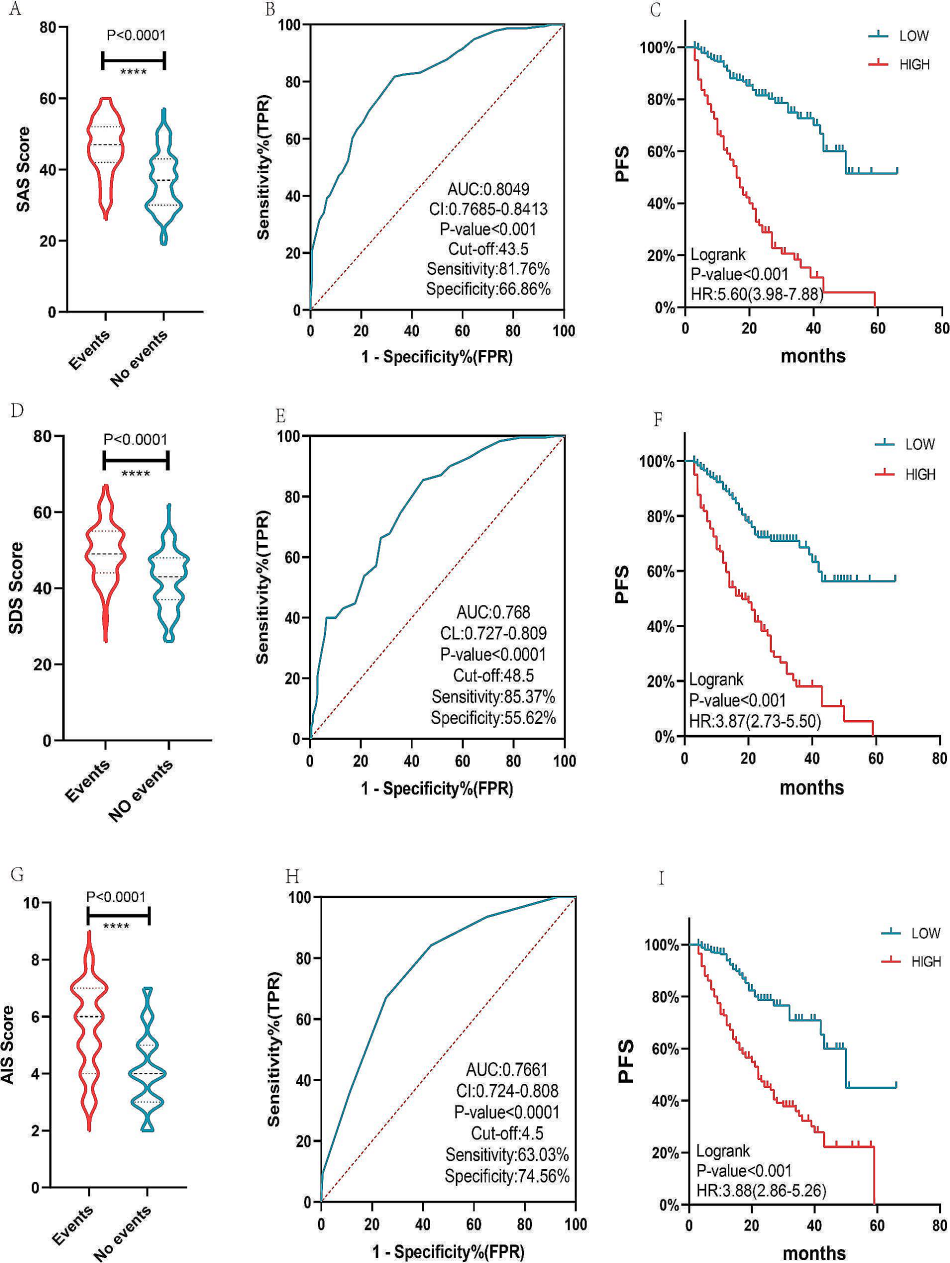
SAS, SDS, AIS score, and postoperative disease progression and survival analysis. SAS, SDS, and AIS scores were statistically different between patients whose disease progressed and those whose disease did not progress (**A**, **D**, and **G**), ROC curve analysis revealed that SAS, SDS, and AIS all had a high ability to differentiate prognosis (**B**, **E**, and **H**). Kaplan-Meier survival analysis indicates that the prognosis of the high SAS, SDS, and AIS score group is significantly worse than the low group. (**C**, **F**, **I**)

According to the results of the study, SAS, SDS, and AIS may be used to differentiate the prognosis of patients. There were statistically significant differences in the SAS, SDS, and AIS scores between patients with and without PD events. The AUC were: 0.8049 (0.7685–0.8613), 0.768 (0.727–0.809), and 0.7661 (0.724–0.808), with cut-off values of 43.5, 48.5, and 4.5 respectively. Kaplan-Meier analysis by grouping SAS, SDS, and AIS by cut-off values showed statistically significant differences between the two groups, logrank test *P* < 0.001, hazard ratio (HR) values of 5.6 (3.98–7.88), 3.87 (2.73–5.5), and 3.88 (2.86–5.26) (Fig. 1).

### Screening of observational indices

**Fig. 2:**
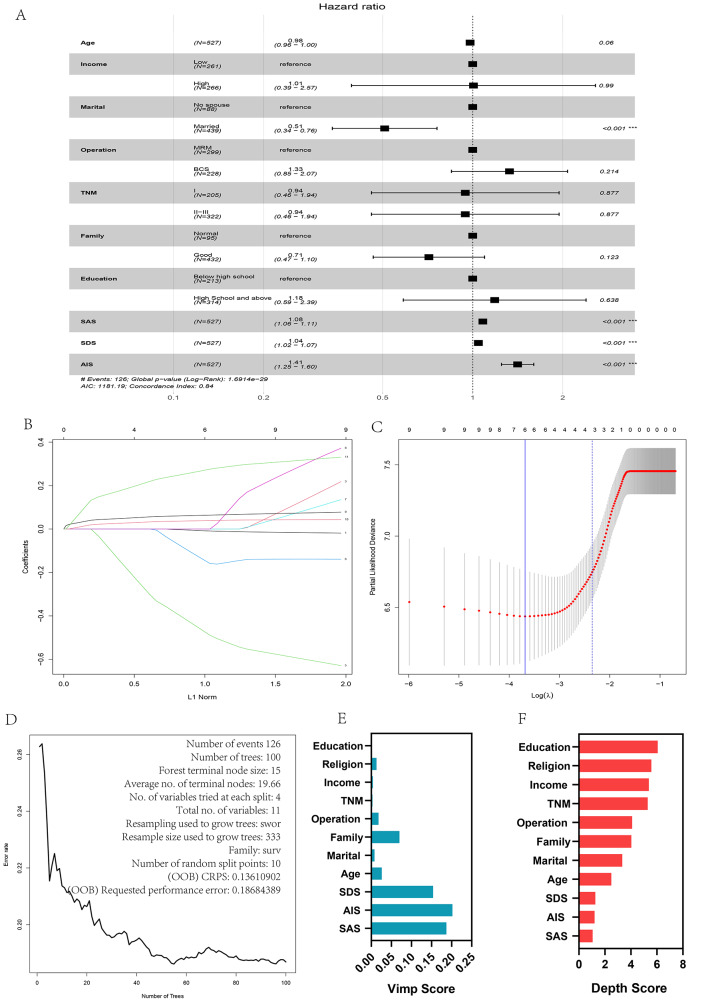
Variable filtering. Forest plots incorporating Cox regression analysis of all variables showed that marital status, SAS, SDS, and AIS scores were significant variables (*P* < 0.05) (**A**). Lasso regression analysis and cross-lasso regression. Lasso regression analysis yields an optimal model incorporating SAS, SDS, AIS scores (**B**, **C**). Random forest model screening variables (**D**, **E**, **F**)

In this study, we intended to develop a prediction model based on the relevant data available at the initial treatment stage and investigate the methods that could guide the accurate implementation of a multidisciplinary continuous care model. These variables included age (years), income, surgical method, marital status, family support, educational background, religion, SAS, SDS, AIS, and prognostic indices. Variance inflation factors (VIF) were calculated for each factor in the multivariate analysis, and it was discovered that the VIFs of age, income, family support, and SAS were all greater than five points, which may be attributed to collinearity among related variables. A lasso analysis was conducted to reduce high-dimensional data, with correlation factors evaluated as the optimal predictive features [13, 14] and a lasso regression model was used to eliminate features with non-zero coefficients [15]. Significant variables were identified using single-factor analysis and multi-factor analysis to create model 1, lasso regression and cross-lasso regression analysis to create model 2, random forest calculation method to create model 3, stepwise regression method (backward method) to create model 4, and including all variables for Cox regression to include significant variables to create model 5 (Table 2; Fig. 2).

**Table 2:** Model comparison, validation and selection

	Age	Income	Operation	Marital	TNM	Family	Education	Religion	SAS	SDS	AIS
Model 1	●			●					●	●	●
Model 2									●	●	●
Model 3	●					●			●	●	●
Model 4	●			●				●	●	●	●
Model 5	●			●					●	●	●

Model1 is the inclusion of variables with *P* < 0.05

Model2 is obtained by cross-lasso regression, resampling according to a 10-fold scheme, and inclusion of variables with non-zero coefficients by regularizing the coefficients

Model3 is the optimal tree obtained by random forest

Model4 is the variables obtained by the stepwise regression method (backward method) with the red pool information criterion

Model5 is the inclusion of all variables for multivariate analysis without univariate analysis, adjusting for all confounders and including variables with *P* < 0.05

### Model validation, comparison, and screening

**Fig. 3:**
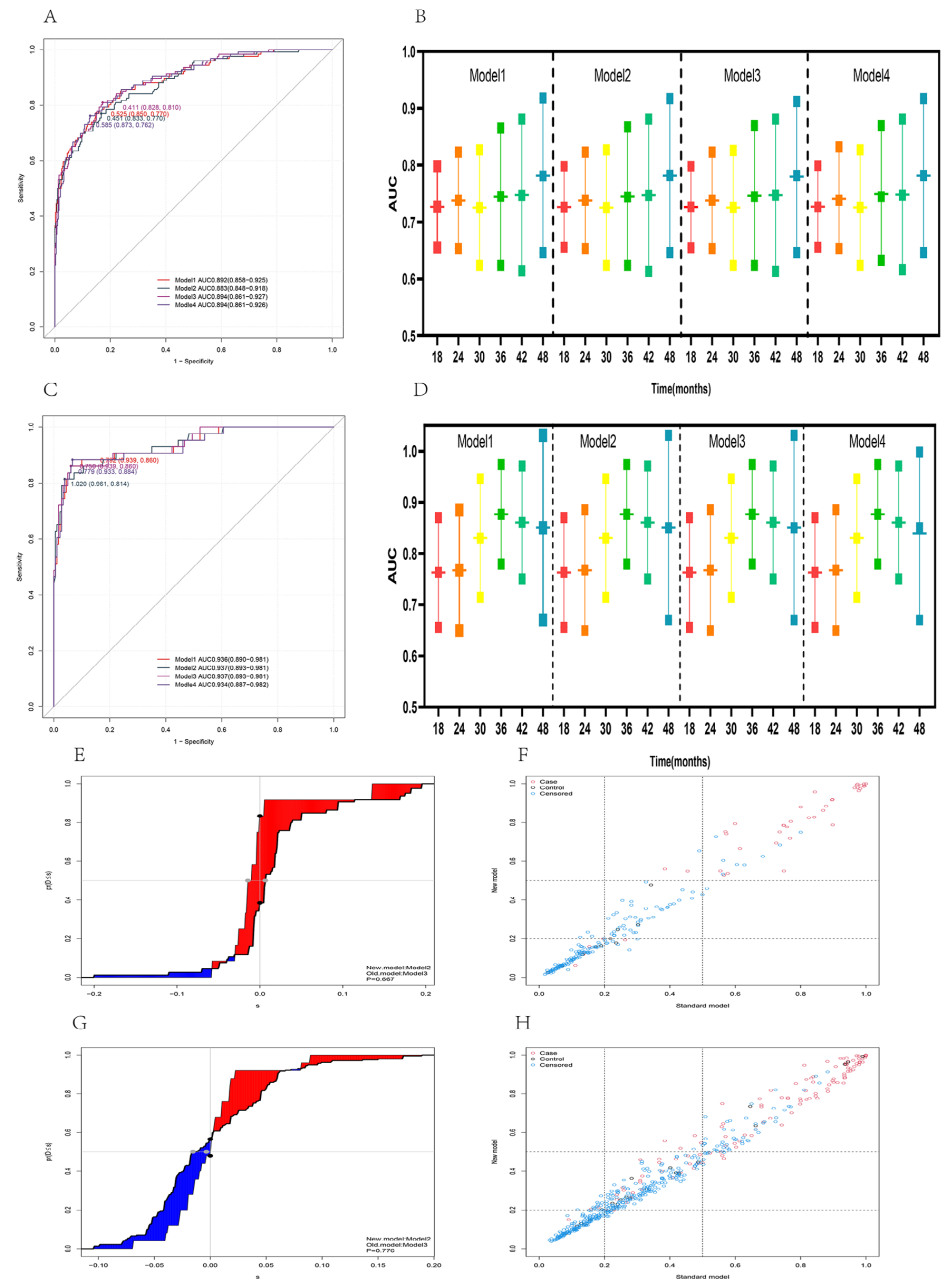
Model comparison, validation, and selection. Comparison of ROC curves of multiple models in the training dataset (**A**); comparison of the area under the time-dependent ROC curve of multiple models in the training dataset (**B**); comparison of ROC curves of multiple models in the validation dataset (**C**); comparison of the area under the time-dependent ROC curve of multiple models in the validation dataset (**D**); IDI analysis of model 2 and model 3 showed no statistically significant difference between the two in both the training and validation datasets (**E**, **G**); NRI analysis of model 2 and model 3 revealed no statistically significant difference between the two in both the training and validation datasets (**F**, **H**)

Five models were developed by screening significant variables into the model using different methods, where model 1 and model 5 were identical, indicating that the variables with *P* < 0.05 were included in the multi-factor analysis by first conducting a one-way analysis, which was consistent with the effect of including all variables directly into the multi-factor analysis in this study; model 5 is not discussed here. The ROC was plotted in the training and validation sets, to investigate the AUC values of different models. This yielded 0.892 (0.858–0.925) for model 1, 0.883 (0.848–0.918) for model 2, 0.894 (0.861–0.927) for model 3, and 0.894 (0.861–0.926) for model 4 in the training set, and 0.936 (0.890–0.98) for the validation set, respectively. 0.981), 0.937 (0.893–0.981), 0.937 (0.893–0.981), and 0.934 (0.887–0.982), respectively. The models demonstrate improved prediction performance in both the training and validation sets, however, the AUC is higher in the validation set than in the training set, probably due to the higher distribution of emergent events in the validation set than in the training set. The prediction performance of different models at different time points was further analyzed using time-dependent ROC curves, and it was found that model 2 had the least number of independent variables, whereas model 3 had the highest prediction performance, The two models were analyzed using NRI and IDI, and there was no statistically significant difference between them in either the training set or the validation set. Therefore, model 2, which incorporated fewer variables, was selected as the subject of a follow-up study to investigate its potential use as a clinical prediction model (Fig. 3).

### Model testing and visualization

**Fig. 4:**
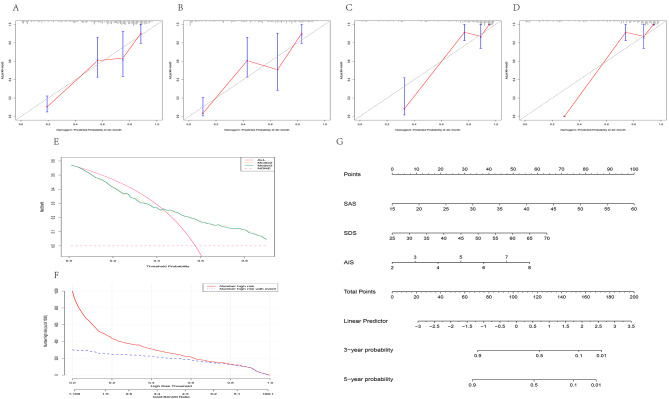
Model testing and visualization. Calibration curves of model 2 in the training dataset for months 40 and 48 (**A**, **B**); calibration curves of model 2 in the validation dataset for months 40 and 48 (**C**, **D**). DCA curves and CIC curves of model 2 in the full data set (**E**, **F**). Nomogram for model 2 (**G**)

The calibration curves were plotted for model 2 at two time points of 40 and 48 months, respectively, and for the training and validation sets of even data sets. It was discovered that although model 2 did not overlap the standard line, fluctuating above and below it, the overall predictive stability was fair, with DCA and CIC curves, indicating that intervention in patients at higher risk may result in clinical benefit, while intervention in patients at lower risk interventions is likely to increase medical consumption. This indicates the need for selective clinical interventions, including interventions in multidisciplinary models of care. Figure 4 depicts a nomogram, which is a visual representation of the integration of multiple predictors based on the multivariate regression analysis, in which a value was assigned, and a scaled line segment was drawn for each index to make it convenient for clinical use [16, 17]. Nomogram was used to predict linear survival probabilities at both 36 and 60 months. The CIC map analysis revealed a convergence of the two curves when the risk value reached 0.6. Specifically, at this threshold, the model’s effectiveness at 36 months exhibited the following metrics: Sensitivity: 0.8047, Specificity: 0.8124, Positive Predictive Value: 0.5551, Negative Predictive Value: 0.9347, Prevalence: 0.2253, Detection Rate: 0.1813, Detection Prevalence: 0.3267, Balanced Accuracy: 0.8086, Precision: 0.5551, Specificity: 0.8124, F1 Score: 0.6570, and Recall: 0.8047. Similarly, at a risk value of 0.6 and 60 months, the model’s performance was as follows: Sensitivity: 0.8402, Specificity: 0.7556, Positive Predictive Value: 0.5000, Negative Predictive Value: 0.9421, Prevalence: 0.2253, Detection Rate: 0.1893, Detection Prevalence: 0.3787, Balanced Accuracy: 0.7979, Precision: 0.5000, Specificity: 0.7556, F1 Score: 0.8402, and Recall: 0.8402.

### Convert model 2 to a scoring system and validate

**Fig. 5:**
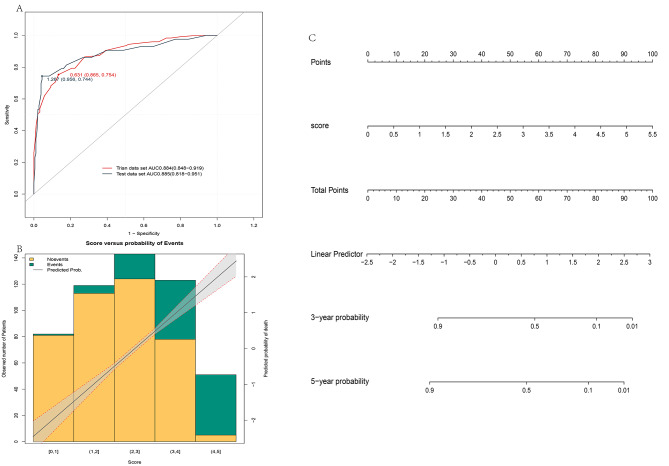
Validation and visualization after model 2 transformation scoring. ROC curves for transformed model 2 scores in the training and validation datasets; (**A**). Analysis of the likelihood and correlation of disease progression in different score areas (**B**). Nomogram of model 2 after transforming the score (**C**)

While the predictive efficacy and stability of Model 2 developed with the continuous scores of SAS, SDS, and AIS are higher, the continuous scores are inconvenient for clinical use. We analyzed the relationship between the observed variables and the outcome variables (see Appendix 2 for details), excised the continuous variables into several intervals based on the change trend, and assigned values to each interval using Cox regression analysis (see Table 3 for details). We then aggregated the continuous variables to obtain the total score, performed Cox regression analysis on the total score, and analyzed the relationship between the total score and the outcome. We discovered that in both the training and validation sets, the total score had better predictive efficacy, with AUC values of 0.884 (0.848–0.919) and 0.885 (0.818–0.951), respectively. As the score increases, patients have an increased risk of disease progression. We also plotted the column line graphs to visualize our results (Fig. 5).

**Table 3:** The score of each interval

SAS	[19, 32]	[32, 42]	[42,50]	[50,60]	
Score	0	1.2	1.8	2.2	
SDS	[26, 35]	[35, 40]	[40, 48]	[48,57]	[57,67]
Score	0	0.7	1	1.7	2
AIS	[2, 4]	[4, 6]	(6, 8]		
Score	0	0.9	1.2		

## Discussion

The incidence rate of breast cancer is rising annually. This disease poses a significant threat to the health of women and has also been linked to depression and sleep disorders. Approximately 30–40% of patients diagnosed with breast cancer and undergoing treatment, experience depression [18]. Group psychological intervention is beneficial to the psychological adjustment of patients with breast cancer [19], and conventional nursing models usually provide conventional psychological interventions for patients receiving chemotherapy. Although initial results have been achieved, the overall effect is not optimal [20]. Consequently, it is critical to develop a new nursing model.

There are examples of multidisciplinary continuous models improving the negative mood and quality of life of patients with cancer with a variety of tumors [21, 22]. The importance of multidisciplinary continuous models is increasingly emphasized as they can improve both negative mood and sleep disorders, thereby improving patient prognoses [23]. However, these nursing models require a combination of multiple disciplines, which is not only quite difficult but also demands significant staffing and material resources, hence it is worthwhile to consider how to achieve more accurate predictions and implementation.

These issues were discussed in this study, and the results demonstrated that SAS, SDS, and AIS scores could be used to differentiate the prognoses of patients. SAS, SDS, and AIS scores differed in the group of patients who experienced postoperative disease progression versus those who did not; the differences were statistically significant, and the ability to differentiate prognosis was high. The AUCs were: 0.8049 (0.7685–0.8613), 0.768 (0.727–0.809), and 0.7661 (0.724–0.808), with cut-off values of 43.5, 48.5, and 4.5 respectively. Kaplan-Meier analysis by grouping SAS, SDS, and AIS by cut-off values revealed statistically significant differences between the two groups, logrank test *P* < 0.001, HR values of 5.6 (3.98–7.88), 3.87 (2.73–5.5), and 3.88 (2.86–5.26), respectively which is consistent with the findings of Li et al. [24]. This may be due to the fact that depression and sleep disorders can reduce immune capacity and increase the possibility of discontinuing or resisting treatment. Nomograms are considered easier and more accurate for clinical evaluation than multi-index combined prediction models, which have been extensively used in clinical practice. Both methods can improve clinical decision-making from multiple perspectives [25]. In clinical practice, an effective method for distinguishing the risk of adverse mood and sleep difficulties impacting breast cancer progression is lacking. The shortage in human and material resources further complicates the implementation of a comprehensive, whole-process multidisciplinary nursing model for all patients. This underscores the need for more precise differentiation and targeted intervention strategies. In our preliminary study, we established a non-inferior study based on the prediction model [26]. Patients were categorized, interventions were administered to high-risk groups, and the overall benefits for the entire population were assessed. While the results did not fully meet expectations, the insights gained from the overall impact provide valuable guidance for our ongoing clinical efforts.

We evaluated the independent variables using single-factor analysis and multi-factor analysis using lasso regression and cross-lasso regression, respectively. Lasso regression analysis, random forest, stepwise regression method (backward method), and Cox regression for all variables were used to establish the model, and comparative analysis of the model was performed to identify a model suitable for clinical use, i.e., an accurate and simple to use model. In the subsequent analyses, we found that the predictive efficacy and stability of the model were good, which also suggested that emotional and sleep factors are likely to be significant factors independent of the condition, consistent with previous studies [10, 27, 28] but this requires further investigation.

Furthermore, the DCA and CIC curves indicate the need for accurate prediction and differentiation of patients, as arbitrary clinical interventions (such as interventions for psychological factors and multidisciplinary combined care model interventions) may lead to unnecessary increases in medical consumption among the low-risk group. The clinical workload is extremely heavy, and continuous values can generate a substantial amount of additional work. Nomogram analysis was used to predict linear survival probabilities at both 36 and 60 months. The CIC map analysis revealed a gradual convergence of the two curves at a risk value of 0.6. At 36 months, the model demonstrated a performance with a Balanced Accuracy of 0.8086, Precision of 0.5551, Specificity of 0.8124, F1 score of 0.6570048, and Recall of 0.8047337. Similarly, when the risk value is 0.6, the model’s performance at 60 months includes a Balanced Accuracy of 0.7979, Precision of 0.5000, Specificity of 0.7556, F1 score of 0.8402, and Recall of 0.8402. Consequently, patients were categorized with a risk value exceeding 0.6 as high-risk individuals with an elevated likelihood of disease progression. Our primary focus was on enhancing positive predictions, mitigating the risk of underestimating disease progression in high-risk individuals, and seeking opportunities for clinical intervention. Through data analysis, we attempted to transform continuous variables according to the characteristics of the data distribution. This transformation was validated and found to be feasible. We attempted to develop a scoring system for effective prediction of disease progression. Results from the validation set showed that the scoring system meets the prediction needs, suggesting it could be used to gauge the urgency of a follow-up intervention.

While explaining the findings of our study, we should also explain the limitations of our study. First, this is a single-center regression study, which has a bias in the inclusion of subjects, and second, it does not include, for example, patients’ current emotions, personal opinions and coping with social expectations. Among the included variables, we included income, surgical treatment plan, stage, marital and family support, as well as the patient’s education and religious belief, but did not include more data related to social support, which related literature suggests has an impact on breast cancer prognosis [29, 30]. These factors affect the generalization ability of our model. Thirdly, some patients lost follow-up after the end of the study follow-up period and were unable to update further survival events, but we can further explore the rule of these patients from the K-M survival curve, although there are certain shortcomings in this study. However, it does not affect the sharing of thinking and problem-solving methods elaborated in our research, but in clinical application, further research is still needed to verify.

## Conclusion

In this retrospective study, we investigated the correlation between disease progression in breast cancer and its related variables. This was visualized using a nomogram to guide clinical nursing decision-making, accurately implement the multidisciplinary continuous nursing model, and further reduce depression and sleep disorders in patients with breast cancer, thereby helping to reduce PD events. Due to the retrospective nature of the study, prospective clinical validation was not conducted; therefore, additional clinical validation is required.

### Electronic supplementary material

Below is the link to the electronic supplementary material.

Supplementary Material 1

## Data Availability

All data generated or analysed during this study are included in this article. Further enquiries can be directed to the corresponding author.

## Abbreviations

AIS: Athens insomnia scale
CIC: Clinical impact curve
DCA: Decision curve analysis
NCCN: National Comprehensive Cancer Network
NRI: Net reclassification index
NRI: Net reclassification index
PD: Progressive disease
ROC: Operating characteristic
SAS: Self-rating anxiety scale
SDS: Self-rating depression scale
TNM: Tumor/number/metastasis
VIF: Variance inflation factors
