# Supplementary material for: Development and validation of a nomogram model of depression and sleep disorders and the risk of disease progression in patients with breast cancer
Source: BMC Womens Health. 2024 Jul 3;24:385. doi: 10.1186/s12905-024-03222-9 (PMC11220980; doi:10.1186/s12905-024-03222-9)

Appendix 1. Baseline indexes

|  | All data set | Train data set | Test data set |
| --- | --- | --- | --- |
| Characteristic | N = 750^1^ | N = 527^1^ | N = 223^1^ |
| Age | 62.9(7.6) | 62.6(7.9) | 63.4(6.7) |
| Income |  |  |  |
| Low | 394(53%) | 261(50%) | 133(60%) |
| High | 356(47%) | 266(50%) | 90(40%) |
| Operation |  |  |  |
| MRM | 420(56%) | 299(57%) | 121(54%) |
| BCS | 330(44%) | 228(43%) | 102(46%) |
| TNM |  |  |  |
| I | 304(41%) | 205(39%) | 99(44%) |
| II-III | 446(59%) | 322(61%) | 124(56%) |
| Marital |  |  |  |
| No spouse | 130(17%) | 88(17%) | 42(19%) |
| Married | 620(83%) | 439(83%) | 181(81%) |
| Family |  |  |  |
| Normal | 128(17%) | 95(18%) | 33(15%) |
| Good | 622(83%) | 432(82%) | 190(85%) |
| Education |  |  |  |
| Below high school | 334(45%) | 213(40%) | 121(54%) |
| High School and above | 416(55%) | 314(60%) | 102(46%) |
| Religion | 96(13%) | 73(14%) | 23(10%) |
| SAS | 38.8(9.0) | 38.9(9.1) | 38.6(8.9) |
| SDS | 43.7(7.9) | 43.9(7.7) | 43.3(8.3) |
| AIS | 4.4(1.5) | 4.5(1.5) | 4.4(1.4) |
| Events |  |  |  |
| No events | 581(77%) | 401(76%) | 180(81%) |
| Events | 169(23%) | 126(24%) | 43(19%) |
| Follow-up time  **Median**(**IQR**) | 10(6,17) | 10(6,17) | 11(7,17) |
| ^1^Mean(SD); n(%)；**Median**(**IQR**) | | | |

**Appendix 2:** (A-F) Relationship between SAS, SDS, and AIS scores in the training dataset and validation dataset and outcome variables. (A, B, C) As scores increase in the training set, the risk of disease progression increases for SAS, SDS, and AIS. (D, E, F) As scores increase in the validation set, the risk of disease progression increases for SAS, SDS, and AIS. (G-N) Time-dependent ROC curves for different models in the training and validation datasets. (G, H, I, J) Time-dependent ROC curves for model 1, model 2, model 3, and model 4 in the training set. (K, L, M, N) Time-dependent ROC curves for model 1, model 2, model 3, and model 4 in the validation set.


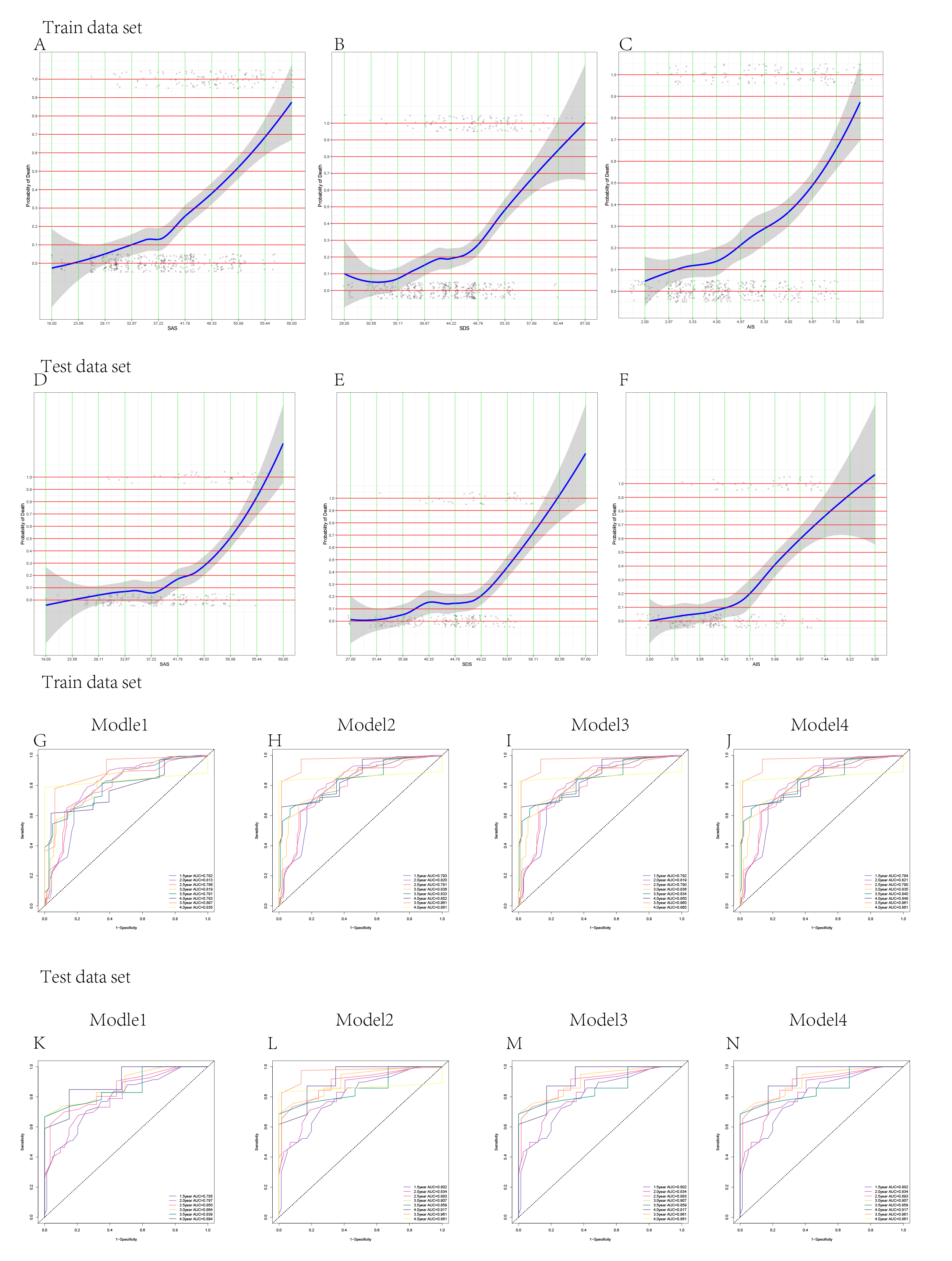

Supplement: Supplementary file 1 — Supplementary Material 1 [file 12905_2024_3222_MOESM1_ESM.docx]
